# Early Language Competencies and Advanced Measures of Mental State Understanding Are Differently Related to Listening and Reading Comprehension in Early Adolescence

**DOI:** 10.3389/fpsyg.2020.00952

**Published:** 2020-06-17

**Authors:** Susanne Ebert

**Affiliations:** Department of Psychology, Norwegian University of Science, Trondheim, Norway

**Keywords:** reading comprehension, listening comprehension, language, theory of mind, metacognitive knowledge, mental state language, longitudinal study

## Abstract

The present study tests a section of the DIET (direct and indirect effects model of text comprehension; Kim, 2017) model and focuses on the relations between early language skills, various facets of mental state understanding, and text comprehension. In a sample of 267 children, I analyzed the relations between language skills (vocabulary, sentence comprehension) at age 3;6, theory of mind (ToM) at age 5;6, mental state language and metacognitive knowledge at age 9;2, and children’s listening and reading comprehension of texts at age 13;7 years. For reading comprehension, results favored a total mediation model that included only direct links from metacognitive knowledge and mental state language to reading comprehension. For listening comprehension, by contrast, a model that also included direct relations from language and ToM in preschool was favored. Metacognitive skills did not mediate the relation between early skills and later text comprehension but, along with mental state language, showed direct relations with reading comprehension beyond listening comprehension. Early language skills showed various indirect relations with later reading comprehension via ToM, mental state language, and listening comprehension, whereas ToM showed only small indirect relations with later reading comprehension via later listening comprehension. These different relations of the various components with later listening in contrast to reading comprehension are discussed.

## Introduction

It is widely known that language plays a major role in the development of reading comprehension (e.g., Lervåg et al., 2009; Dickinson et al., 2010; Ebert and Weinert, 2013). Moreover, language is closely connected to children’s developing understanding and knowledge about mental states and processes (Astington and Baird, 2005a; Ebert, 2015). Again, particularly in recent years, children’s developing understanding of mental states and processes, for example, theory of mind (ToM) and metacognition, have also been discussed as relevant for children’s reading comprehension (e.g., Lecce et al., 2010; Neuenhaus et al., 2011; Atkinson et al., 2017; Ebert, 2020). Against this background, the main aim of the present study was to investigate how children’s early language competencies – along with their developing knowledge and understanding of mental states and processes – are related to their reading comprehension in early adolescence. In addition, I asked whether the relations would be different for listening and reading comprehension and whether early language and mental state knowledge and understanding would show relations with later reading comprehension that could not be totally explained by concurrent listening comprehension (see also Kim, 2017).

Note that in the present study, reading comprehension refers to the comprehension of written texts, whereas listening comprehension refers to the comprehension of orally presented texts. All other (oral) language comprehension measures are called language skills (e.g., vocabulary, sentence comprehension).

### Models of Reading

According to one of the best known models of reading – the simple view of reading (Hoover and Gough, 1990) – reading comprehension is a product of decoding skills and listening comprehension. Decoding refers to the encoding of written material and the ability to read written material fluently, whereas comprehension refers to understanding the meaning behind language, written or oral. Decoding and comprehension depend on each other because without decoding, no comprehension of written text is possible, and without comprehension, the decoding is more or less useless. However, these two processes have different developmental pathways and predictors (Oakhill et al., 2003; Oakhill and Cain, 2012). Thus, whereas phonological information processing skills (e.g., phonological awareness) are stronger predictors of decoding processes such as word reading, (oral) language skills (e.g., vocabulary) are stronger predictors of language comprehension such as listening and reading comprehension of texts (e.g., Ebert and Weinert, 2013; Hjetland et al., 2017; Lervåg et al., 2018).

In the present study, I focused on reading comprehension in early adolescence, when decoding processes play only a minor role in reading comprehension. At this age, reading comprehension (i.e., extracting the meaning behind written language) is only little constrained by decoding processes and thus the contribution of (oral) language skills to reading comprehension is more important (Storch and Whitehurst, 2002; Vellutino et al., 2007; Foorman et al., 2018; Lervåg et al., 2018).

The component skill model (e.g., Oakhill et al., 2006; Oakhill and Cain, 2012) proposes that different aspects of language such as grammar and vocabulary predict later text comprehension (see also Suggate et al., 2018). These different aspects may also be important for text comprehension at different points of development (see Muter et al., 2004; Cain, 2016).

Besides vocabulary and grammar as fundamental language skills, the component skill model also proposes that children’s general cognitive abilities, particularly their working memory, predict later text comprehension (e.g., Muter et al., 2004; Oakhill et al., 2006; Kim, 2017).

However, besides these foundational language and cognitive skills, higher-level skills as for example, integration and interference, knowledge and use of text structure, and comprehension monitoring, play a role in text comprehension (Cain, 2016). Similar to (oral) language skills, it can be assumed that the contribution of higher-order skills becomes more relevant over time for reading comprehension, when decoding is less constraining, but also for text comprehension in general because texts become more complicated. Oakhill et al. (2003), for example, showed that verbal working memory and higher-order skills such as inference skills and comprehension monitoring accounted for unique variance in reading comprehension between the ages of 7 and 9 over and above foundational language skills. However, another longitudinal study showed that even in the preschool years, foundational language skills and higher-order skills such as inferential skills accounted for unique variance in listening comprehension between 4;10 and 5;5 years (Florit et al., 2014).

The DIET (direct and indirect effects model of text comprehension) model differentiates more explicitly how these different foundational and higher-order components are related to text comprehension (Kim, 2017): Foundational cognitive skills (e.g., working memory) are the basis for foundational language skills (e.g., vocabulary), and both foundational cognitive and language skills are necessary but not sufficient for text comprehension. Thus, they might have direct but also indirect effects on text comprehension via higher-order skills (Kim, 2017). Higher-order skills (e.g., inference skills or comprehension monitoring) rely on these foundational skills and help to integrate them so that they can be used to build a situation model. A situation or mental model of the text characterizes successful text comprehension. It refers to a mental representation of the actual meaning behind a text. There are different levels of mental representations: for example, the representation of phrases and sentences as well as the representation of propositions and units. The situation model is the highest level of representation that leads to a meaning-based representation of the situation through the integration of text-based information with prior knowledge (Kintsch and van Dijk, 1978; see also Zwaan, 2016; Kim, 2017).

In the present study, I focused on a section of the DIET model: on language skills as well as higher-order skills that are related to the understanding and knowledge of mental states and processes, and I determined how these are related with one another and to text comprehension. As higher-order skills that are related to the understanding and knowledge of mental states, I refer to three facets of mental state understanding that are theoretically and empirically connected: theory of mind (ToM), metacognitive knowledge, and mental state language (Hughes and Dunn, 1998; Bartsch, 2002; Antonietti et al., 2006; Lockl and Schneider, 2006; Ebert, 2011, 2015).

### Theory of Mind (ToM)

ToM refers to the knowledge and understanding of mental states and processes and more broadly comprises social understanding in general. One main step in children’s ToM development is their understanding of false beliefs between the ages of 3 and 5 (Wellman et al., 2001). It is assumed that when children have developed this understanding that beliefs can be false (i.e., they can change and differ from reality), they have developed a metarepresentational understanding of the mind (Perner, 1991). This understanding may support them in understanding multiple perspectives and psychological causality earlier, more quickly, and more flexibly (Dore et al., 2018). Consequently, having developed a metarepresentational ToM understanding may support children’s text comprehension via inference making skills about an author’s intentions, and characters’ thoughts and feelings (Cain, 2016; Kim, 2017).

However, previous studies investigating the link between ToM and reading comprehension have shown mixed results. Whereas some have reported significant direct effects of ToM on reading comprehension, even after accounting for language skills and listening comprehension (Atkinson et al., 2017; Boerma et al., 2017), others have not found direct links after considering language skills or listening comprehension (Guajardo and Cartwright, 2016; Kim, 2017; Ebert, 2020) or they have found no correlations at all (Lockl et al., 2017). However, there is evidence that suggests that, particularly beyond the preschool years, when more advanced measures of reading and more advanced measures of ToM such as higher-order mental reasoning or the reference to mental states in more complex situations are assessed, ToM is related to reading comprehension (Boerma et al., 2017; Florit et al., 2020). This may be since, (a) advanced reading comprehension is less constrained by decoding, (b) texts are getting more sophisticated, and (c) higher-order reasoning or advanced ToM tasks assess mental reasoning in complex social scenarios that require people to make inferences about mental states. These specific inference skills may, in particular, support children’s text comprehension. However, other advanced aspects of mental state understanding that are related to ToM may also support children’s reading comprehension. Two such advanced aspects of children’s understanding of mental states and processes are considered in the present study: children’s mental state language and children’s metacognitive knowledge.

### Mental State Language

Mental state language refers to language that is used to express mental states and processes (Bretherton and Beeghly, 1982; Antonietti et al., 2006; Olson et al., 2006). This includes, in particular, terms that are used to describe mental states such as desires, intentions, or knowledge (e.g., “want,” “belief,” “knowledge,” “memory”). The knowledge of such specific terms usually requires an understanding of these concepts (e.g., Gopnik and Meltzoff, 1986). This leads to the conclusion that the comprehension of mental terms is an expression of children’s understanding of ToM (see also Astington and Pelletier, 2005). Indeed, correlations between children’s ToM and their comprehension of mental terms in the preschool years have been reported (e.g., Moore et al., 1990; Lockl and Schneider, 2006; Howard et al., 2008). Moreover, the understanding and use of more complex mental state terms in school (e.g., “assume,” “conclude”) is seen as an advanced ToM (Schwanenflugel et al., 1998; Astington and Pelletier, 2005; Antonietti et al., 2006; Olson et al., 2006; Peterson and Slaughter, 2006). Lecce et al. (2010), for example, used school-aged children’s mental state words produced on a writing task as an indicator of children’s mental state knowledge. However, we do not yet know how ToM in preschool is related to the advanced comprehension of mental state language.

Astington and Pelletier (2005) assumed that learning from texts requires the comprehension of mental state terms. Mental state language is seen as a tool that supports thinking and reasoning about representations (see also Olson et al., 2006) and that could help children compare and integrate text-based information with prior knowledge and build a situation model of the text. Thereby, mental state terms may help children understand what the authors meant by their words. This idea was supported by a study that showed that children trained in conversation about the mind, including a lot of mental state words, were more accurate when making mental-state attributions (Bianco et al., 2016).

However, although there are theoretical assumptions about the relation between mental state terms and text comprehension, not much empirical research has investigated the relation between mental state language and reading comprehension. Astington and Pelletier (2005) report that the comprehension of mental state terms made a small but significant contribution to the prediction of change in reading from the first to the second grade. By contrast, Lecce et al. (2010) found a significant relation between children’s use of mental state words in a writing task and their reading comprehension only for 4th graders but not for 2nd graders.

### Metacognitive Knowledge

Metacognition is broadly defined as knowledge about knowledge. On the one hand, it refers to children’s factual knowledge about cognitions (e.g., about memory, strategies, comprehension), and on the other hand, it refers to the controlling and monitoring of mental states and processes (Flavell et al., 2002). In the present study, I focused on factual knowledge about cognition because ToM in preschool has been shown to be a precursor of later metacognitive knowledge (Lockl and Schneider, 2007; Lecce et al., 2014; Ebert, 2015). There is also some evidence that ToM is related to metacognitive knowledge beyond the preschool years. Thus, Lecce et al. (2010) showed that even after they controlled for verbal abilities, 10-year-old children’s advanced ToM measured via a social scenario test was related to their metacognitive knowledge about reading concurrently and about one year later.

From a theoretical perspective, knowledge about cognitive processes and particularly knowledge about effective learning strategies should be associated with children’s learning outcomes and thus also with their reading comprehension. For example, even though knowledge about strategies does not necessarily lead to the use of a strategy whenever indicated, children who have rich knowledge about strategies know at least how and when to use a strategy and probably use strategies more often and more appropriately (Artelt and Schneider, 2015). In this vein, various studies that have included children in primary and secondary school have shown that children’s metacognitive knowledge is related to their reading comprehension (e.g., Lecce et al., 2010; Artelt and Schneider, 2015; Edossa et al., 2019; Soto et al., 2019). However, these studies have mostly been cross-sectional and were therefore not able to consider developmental relations or other earlier variables that are related to metacognitive knowledge and reading comprehension, such as language skills.

### Language Skills

ToM, mental state language, and metacognitive knowledge might all contribute to children’s text comprehension. Moreover, according to the DIET model, foundational language skills might also be indirectly related to text comprehension via ToM, mental state language, and metacognitive knowledge. Given that in the preschool years, children’s language skills predict ToM (Astington and Baird, 2005b; Milligan et al., 2007), language skills may be likely to show indirect effects via ToM. Indeed, Kim (2017) was able to demonstrate this indirect relation in 2nd graders using second-order false belief tasks, i.e., tasks that assess the understanding of false beliefs about mental representations. Moreover, given that ToM is theoretically closely related to mental state language and metacognitive knowledge (see above), I further expected to find that language skills also have indirect relations with text comprehension via ToM which then is related to mental state language and metacognitive knowledge. Furthermore, given that the comprehension of mental terms is also a specific language skill and given the evidence that metacognitive knowledge is also predicted by language skills (Lecce et al., 2010; Ebert, 2011, 2015), I further expected to find that foundational language skills also have indirect relations with text comprehension via mental state language and metacognitive knowledge.

### Listening and Reading Comprehension

In extending the DIET model, which is a general model of text comprehension, the DIER (direct and indirect effects model of reading comprehension) model integrates the ideas of the simple view of reading and differentiates between listening and reading comprehension (Kim, 2017). As children get older and decoding processes are less likely to constrain their reading comprehension, the relation between reading comprehension and listening comprehension becomes stronger (Foorman et al., 2015). Thus, in advanced reading comprehension, listening comprehension explains most of the variance in children’s reading comprehension. In this vein, Kim (2017) showed that listening comprehension completely mediated the relation between higher-order skills and reading comprehension.

However, although according to the simple view of reading the explanatory mechanism behind reading and listening comprehension should be similar in advanced reading comprehension, specific effects of reading and listening comprehension are possible. Even in the later stages of reading development, there are variables, such as higher-order skills, that might affect reading comprehension beyond listening comprehension (see for example Kirby and Savage, 2008; Kim, 2015; Silva and Cain, 2015).

In particular, metacognitive knowledge might show direct relations with reading comprehension that are not explained by listening comprehension. For instance, children can use their knowledge about reading strategies only for reading but not for listening comprehension (e.g., the knowledge that it is useful to reread a challenging text passage or to underline essential words or sentences), whereas they can often use their knowledge about listening comprehension for reading and listening comprehension. This means, children may have fewer opportunities to actively engage in strategy use in listening comprehension (see also Kirby and Savage, 2008). Thus, I hypothesized that metacognitive knowledge would be more strongly related to reading comprehension than to listening comprehension and would also have a direct relation with reading comprehension after listening comprehension was controlled for.

By contrast, for ToM and mental state language, I hypothesized that listening comprehension would completely mediate the relation to reading comprehension as both skills should be important for text comprehension, no matter whether the text is written or oral.

### The Present Study

The main aim of the present study was to test a section of the DIET model with a special focus on the relations between language, facets of mental state understanding, and text comprehension. Therefore, I extended the DIET model by adding metacognitive knowledge and mental state language as mediators of how the foundational skills and ToM are related to text comprehension. In contrast to other higher-order skills (e.g., inference making), the ways in which these facets of mental understanding are related to text comprehension have yet to be investigated.

**FIGURE 1:**
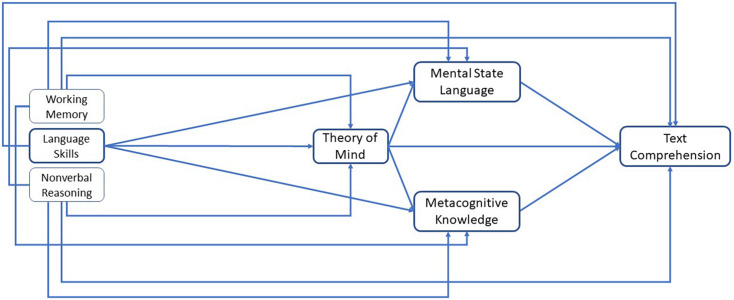
Path model testing a section of the DIET (direct and indirect effects model of text comprehension) specifying the relation between language skills, facets of mental state understanding and text comprehension.

In particular, I investigated the extent to which ToM in the last year of preschool as well as mental state language and metacognitive knowledge in 3rd grade mediate the relations between early language skills at the beginning of preschool and text comprehension in early adolescence. Moreover, I was interested in whether foundational language skills also have direct relations with later text comprehension when higher-order skills that are related to mental state knowledge and understanding are considered. Thus, I specified a model (see Figure 1) for the relations between language skills, the different facets of mental state understanding, and text comprehension based on the DIET model (Kim, 2017). Against the background that ToM is a prerequisite of more advanced facets of mental state understanding such as metacognitive knowledge and mental state language (e.g., Ebert, 2011, 2015), I further expected that ToM might have direct and indirect relations with later text comprehension via those advanced facets of mental state understanding. In addition, besides foundational language skills, I also included foundational cognitive skills (working memory, non-verbal reasoning) as control variables. Particularly working memory is considered in the DIET model and may also be related to later text comprehension (Florit et al., 2009; Kim, 2017).

Kim (2015, 2017) conducted cross-sectional tests of the DIET model in Korean kindergarten children and 2nd graders. However, cross-sectional models do not consider the developmental associations between foundational skills and higher-order skills, although it is assumed that foundational skills precede higher-order skills.

Other studies have considered the link between earlier foundational skills and text comprehension (e.g., Oakhill et al., 2003; Oakhill and Cain, 2012; Florit et al., 2014). However, these studies focused on higher-order skills such as inference skills but did not include measures of mental state language and metacognitive knowledge. Thus, it is not known how they relate along with foundational language skills and ToM to later text comprehension.

Moreover, longitudinal studies in reading development have often focused on the developmental period between (late) preschool and the early school years or on only the early school years (e.g., Roth et al., 2002; Storch and Whitehurst, 2002; Oakhill et al., 2003; Muter et al., 2004). Thus, to further extend previous longitudinal studies that have explored the link between foundational and higher-order skills in relation to text comprehension, I investigated the time period that stretches from early preschool to early adolescence. Although I did not include repeated measures of the same variables and was thus unable to investigate developmental trajectories, I assessed foundational skills that occur earlier in development than higher-order skills. This enabled me to say something about the developmental relations between the variables. However, it is important to mention that data of this type do not allow conclusions to be drawn about the causal effects that earlier variables might have on later ones.

A second aim of the present study was to investigate whether language skills and higher-order variables associated with mental state understanding are differentially related to listening and reading comprehension and whether listening comprehension completely mediates the relations of cognitive and language variables with advanced reading comprehension (see also Kim, 2015, 2017).

Previous longitudinal studies have often focused on either predictors of listening comprehension in preschool aged children (Florit et al., 2011; Lepola et al., 2012; Florit et al., 2014) or on predictors of reading comprehension in school aged children (e.g., Oakhill et al., 2003; Oakhill and Cain, 2012). To extend these studies, I not only investigated a longer developmental period from preschool to early adolescence, but both listening and reading comprehension at the same measurement point in early adolescence. This enabled me to investigate whether these two types of text comprehension have similar earlier predictors. Further, I was also able to analyze whether any of the variables had direct relations with reading comprehension beyond listening comprehension. Based on the DIER model, I included listening comprehension and reading comprehension in one model and expected that listening comprehension would explain most of the direct relations of early language and cognitive skills with reading comprehension (see also Kim, 2017). However, I also expected differential relations of the higher-order skills I investigated in the present study with listening compared to reading comprehension. In particular, I hypothesized that metacognitive knowledge would be more strongly related to reading comprehension than to listening comprehension.

## Materials And Methods

### Participants and Procedure

A subsample of 267 children from a more comprehensive German longitudinal study, who in contrast to the rest of the sample per design were administered measures of ToM at age 5, were part of this study. Besides the ToM measure, various other measures and measurement points from the more comprehensive longitudinal study were included. At the first measurement point of the entire study, which was also Time 1 in the present study, the children had a mean age of 3;6 (*M* = 41.70 months, *SD* = 3.96 months). At the other measurement points included in the present study, the children’s averages ages were 5;6 (*M* = 65.45 months, *SD* = 3.96 months), 9;2 (*M* = 110.58 months, *SD* = 3.81 months), and 13;7 (*M* = 162.81 months, *SD* = 3.70 months).

The children were all born in Germany, and most of them (*n* = 244, 92.1%) had at least one parent who spoke German as her or his mother tongue. For 31 (11.6%) of the children, the primary caregiver was not a native German speaker.

The educational and socioeconomic background (SES) of the sample was diverse. About 20% of mothers had a university degree, whereas most other mothers reported that they had completed vocational training (72%), and a few indicated that they had not had any vocational training (8%). The family’s highest ISEI (HISEI; Ganzeboom et al., 1992), an international index of occupational status, had a mean of 51.86 (*SD* = 15.75) on a scale ranging from 16 (e.g., cleaner, unskilled farmworker) to 90 (e.g., judge in a court of law).

Due to drop-out across measurement points, data were not available from all the children who were included in this sample at all measurement points. At 5;6 years (Wave 5 of the entire study), 39 children (14.6%) had left the study. Another four years later, in 3rd grade (Wave 9 of the entire study), 127 children (47.6%) had dropped out, and in early adolescence (Wave 11 of the entire study) 143 children (53.6%) had left the study. Due to illness or refusal to take part in the actual testing at a certain measurement point numbers of participants were further reduced. Table 1 provides an overview of the number of children for whom I had valid data on each measure at the different measurement points. Especially after the children began school, the drop-out rate was high. However, Little’s MCAR test was not significant [χ^2^(226) = 236.72, *p* = 0.30]. This suggests that the data were missing completely at random, and thus, the analyses would probably not lead to biased estimates (Graham, 2003; Enders, 2013). However, I additionally checked for whether the 116 children who had valid reading comprehension data in early adolescence differed on the central variables from the children who did not have valid reading comprehension data in early adolescence. The children did not differ in age, *t*(255) = -0.21, *p* = 0.83, cognitive and language skills, *F*(3, 240) = 1.86, *p* = 0.14, or language background, χ^2^(2) = 3.75, *p* = 0.15, at Time 1. However, the families of the children who left the study had a lower socioeconomic status (HISEI), *t*(264) = -2.30, *p* = 0.02, than the families of the children who remained in the study.

**TABLE 1:** Descriptive statistics for child variables.

	***N***	***M***	***SD***	**Min**	**Max**
**Working Memory (3;6 Years)**					
Digit span (K-ABC)	245	3.13	2.55	0	9
Hand movements (K-ABC)	246	3.17	2.21	0	10
**Nonverbal Reasoning**					
Analogies (SON)	256	6.13	2.42	0	12
Categories (SON)	242	5.69	2.04	0	11
**Language Skills (3;6 Years)**					
Receptive vocabulary (PPVT)	255	29.74	15.78	0	89
Receptive grammar (SC)	254	11.01	4.27	0	19
**Higher-Order Skills**					
Theory of mind (5;6 years)	220	2.98	1.56	0	5
Metacognitive knowledge (9;2 years)	103	7.23	2.60	0	13
Mental state language (9;2 years)	103	8.63	2.06	4	13
**Text Comprehension (13;7 Years)**					
Listening comprehension (DELKO)	115	19.64	3.02	9	24.50
Reading comprehension (NEPS)	116	21.66	6.04	8.50	31.75

*PPVT, Peabody Picture Vocabulary Test; SC, Test for Sentence Comprehension; K-ABC, Kaufman Assessment Battery for Children; SON, Snijders-Oomen Non-verbal Intelligence Test; DELKO, Test for listening text comprehension; NEPS, Test for reading text comprehension.*

The comprehensive study was funded by the German Research Foundation, and compliance with ethical standards was approved by the German Research Foundation. Appropriate consent to take part in this study was obtained from parents, and all the information they provided was voluntary. For the preschool age children, the testing of the children took place in the children’s preschools in a quiet room with only a trained research assistant. At every measurement point in preschool, children took part in three sessions lasting about 30 min, where they received various tests in a standardized order. In primary school, the testing was administered by two trained research assistants in children’s schools, where other children were taking the test at the same time in the same room. In early adolescence, the children were again tested at home by a trained research assistant. The children always had the opportunity to withdraw from testing at any time, and they were given a small gift (e.g., a pen) after each test session. Parents also received a small gift after they had given interviews in their homes, during which we gathered background information and other information about parenting and educational practices.

### Measures

#### Time 1 (age 3;6)

##### Language skills

The children completed a German research version of the PPVT-R (Dunn and Dunn, 1981) as a measure of receptive vocabulary and the sentence comprehension (SC) subtest of the German Language Development Test for 3–5-year-old children (SETK 3-5; Grimm, 2001) as a measure of receptive grammar.

The PPVT consists of sets of 12 items (except the last set, which has 7), and each item shows 4 pictures. For each item, the research assistant read a word, and the children chose the corresponding picture (max = 175).

On the SC test, the children listened to sentences that varied in grammatical complexity. For the first 9 items, children were presented 4 pictures and asked to choose the one that corresponded to the sentence they had just heard. For the next 10 items, children followed instructions that were given in a sentence (e.g., “Put the blue pen under the bag”).

A sum score for language skills was created by z-standardizing and averaging the two language scores. The correlation between receptive vocabulary and sentence comprehension was *r*(254) = 0.68.

##### Working memory

Working memory was assessed with two memory span tests from the German version of the Kaufman Assessment Battery for Children (K-ABC; Melchers and Preuß, 2003).

On the Digit Span test, the children were required to repeat a sequence of digits verbally presented by a research assistant.

On the Hand Movement test, children were asked to repeat sequences of three different hand movements that were performed by a research assistant.

Both tests include 12 items grouped in sets of different lengths (2–5 digits and 2–4 hand movements). The correlation between the subtests was *r*(245) = 0.52. Scores were standardized and averaged.

##### Non-verbal reasoning

Children’s non-verbal reasoning was measured with the Analogies and Categories subtests from the SON-R 2^1/2^-7 (Tellegen et al., 2005). These subtests evaluate children’s non-verbal reasoning while they are required to infer sorting and classification principles from picture cards or in abstract materials of various shapes and colors (max = 17 for Analogies, max = 15 for Categories). The correlation between the subtests was *r*(245) = 0.52; *p* < 0.05. Test scores were standardized and averaged for a total score on non-verbal reasoning.

#### Time 2 (age 5;6)

##### Theory of mind

Children completed one first-order and one second-order ToM task. The first-order ToM task was a false belief task with unexpected content (based on Perner et al., 1987). The second-order ToM task was the birthday puppy story developed by Sullivan et al. (1994). Both tasks were acted out with small figures and toys by the research assistant.

For the first-order task, the children were shown a peanut box and asked what they thought was inside. The box unexpectedly contained a ball that was shown to the children and put back into the box. After making sure that they understood that a ball was in the box and not peanuts, a naive protagonist (P1) arrived, and the children were asked the false belief question (“What does P1 think is in the box?”) and a control question (“Did P1 look inside the box?”). Children received credit for the false belief question only if they answered the control question correctly. After P1 left the scene, the children were asked a second test question about their own beliefs (“Before you looked inside the box, what did you think was inside?”). Thus, the children could earn two points for the first-order false belief task.

For the second-order task, the children listened to a story about a boy who had seen his actual birthday present (a dog) unbeknownst to his mother. They were given three test questions: One first-order question (“Does Mum know that Peter saw the dog?”) and one second-order knowledge access question (“When Grandma calls and asks if Peter knows what his present is, what will Mum say?”) as well as one second-order false belief question (“What present will Peter’s Mum tell Grandma that Peter thinks he is getting?”). If the children passed two control questions to make sure that they followed the story plot, they obtained one point for each correct answer to the test questions (max = 3).

The scores from the first-order and second-order task were correlated [*r*(123) = 0.29, *p* < 0.01] and summed for a total ToM score.

#### Time 3 (age 9;2)

##### Mental state language

To assess the children’s comprehension of mental state language, we developed a test based on an instrument cited by Astington and Olson (1990) and Olson et al. (2006). On this test, children listen to 14 brief stories (see the example in the Appendix). At the end of each story, a protagonist says or thinks something, and children are required to decide which of three presented mental verbs can best be substituted for the verb “think” or “say” (e.g., infer, ensure) in the given story. Children earned one point for each correct chosen verb. Cronbach’s alpha was 0.60.

##### Metacognitive knowledge

To measure the children’s metacognitive knowledge, we used a metacognitive knowledge test that we developed within the more comprehensive longitudinal study. It consists of 14 multiple-choice items, which were to some extent taken or adapted from other studies (for more information, see Haberkorn et al., 2014). For each item, the children listened to a verbally presented memory, comprehension, or learning problem and had to judge which of two or three presented alternatives would probably lead to the best performance or whether the performances would be equal. Children received one point for every correct answer. Cronbach’s alpha was 0.58.

#### Time 4 (age 13;7)

##### Listening comprehension

The listening text comprehension task comprises 6 stories (each with approximately 100–150 words). The stories on this paper-and-pencil test were adopted from the DELKO project (Marx and Stanat, 2009) and vary in the complexity of their vocabulary and syntax. Some stories take place in everyday contexts (e.g., a conversation in a supermarket), whereas some are more informational (e.g., a text about a rare animal). After listening to each story twice, children are asked 3–5 multiple-choice and open-ended questions (25 questions in total). These questions require the children to recall or compare information from the story or to make inferences. Partially correct answers are given 0.5 points. A second rater coded about 22% of the answers, and interrater reliability was good to excellent (intraclass correlation coefficient (absolute agreement) between 0.90 and 0.98; Cohen’s kappa between 0.76 and 0.96). The scores for all items were summed to form a total score (max = 25). Cronbach’s alpha was 0.64.

##### Reading comprehension

The reading comprehension test was developed in the German National Educational Panel Study and was initially developed for 9th graders (NEPS; Gehrer et al., 2012). The paper-and-pencil test consists of five different (informational, commentary, literary, instructional, and advertising) types of texts (approximately 230 words each). Each text is followed by 5–7 questions, mostly multiple-choice questions (1 answer correct out of 4). Other tasks are matching tasks and decision-making tasks. The tasks require children to extract information or to make inferences on the basis of the text. The children had 28 min for the entire test (max = 33 points).

### Data Analysis Strategy

The primary data analysis strategy was structural equation modeling in Mplus 6.0 (Muthén and Muthén, 2012). For all analyses, I included observed variables. I refrained from estimating latent variables to keep the structural equation model simple and the sample-size-to-parameter ratio low. This approach increases the likelihood that the statistical requirements for path models will be met, even when the sample size is small and missing data are estimated (Kline, 2016).

I used a full information maximum likelihood (FIML) approach to account for the missing data. FIML is superior to listwise deletion, pairwise deletion, and similar older methods for handling missing data, especially in small samples and when outcome variables are incomplete (Enders and Bandalos, 2001; Graham, 2003; Enders, 2013), which was the case in our study.

Model fits were evaluated by computing the root mean square error of approximation (RMSEA) and the comparative fit index (CFI) as recommended by Hu and Bentler (1999). RMSEA below 0.08 and a CFI greater than 0.90 were considered to indicate an acceptable model fit. I used the Akaike information criterion (AIC) and the Bayesian information criterion (BIC) to compare the relative fits of different models, and I compared nested models using chi-square difference tests. I always compared the less restrictive model with the more restrictive model. A significant X ^2^-difference test indicates that the less restrictive model fits the data better, whereas a non-significant X ^2^-difference test suggests that the more restrictive model is not significantly different from the less restrictive one. However, it should be favored for reasons of parsimony. Also, the model with smaller BIC and AIC values suggests a better model fit.

To test the DIET model with a special focus on facets of mental state knowledge and understanding, I first ran separate models for reading comprehension and listening comprehension. This was also done to see whether language and the facets of mental state knowledge and understanding would be found to be differently related to listening and reading comprehension. In particular, I analyzed whether a hierarchical structure could be found, whereby foundational cognitive and language skills feed higher-order skills, which mediate the relations between foundational skills and text comprehension. I compared four models with a complete model (Model 1a). In the complete model (Model 1a), I specified all direct and indirect links between the variables. Then I successively removed all the direct links (i.e., set them to zero) and checked for whether the model fit got worse or whether I should favor the model without direct links for reasons of parsimony. In Model 1b, I first removed the direct link between ToM and text comprehension. Although this is a relation between a higher-order skill and text comprehension, I view ToM as a foundational skill in mental state understanding upon which other later developing higher-order skills in mental state understanding build. In Model 1c, I removed the direct links between language skills and text comprehension, and in Model 1d, I removed the direct links between foundational cognitive skills and text comprehension. In a last model, Model 1e, the complete mediation model, I restricted all direct paths to text comprehension to zero, except for those from mental state language and metacognitive knowledge.

To investigate whether listening comprehension mediates the relations of foundational skills assessed earlier and higher-order skills with reading comprehension, I compared three nested models. In the first model (Model 2a), I specified all direct and indirect links. In the second model (Model 2b), I constrained all direct relations of foundational cognitive and language skills as well as ToM as a foundational higher-order skill for more advanced mental state understanding with text comprehension to zero and allowed only direct paths from mental state language and metacognitive knowledge to reading comprehension. In the third model (Model 2c, complete mediation model), I also set those paths to zero. Thus, I specified no direct links between the variables that were assessed earlier and reading comprehension.

In all models, I also controlled for HISEI as a measure of the family’s socioeconomic background as well as of whether the children had a parent with a mother tongue other than German as a measure of the family’s language background. Hence, in preliminary analyses, paths between these control variables and all outcome measures at all measurement points were specified. However, these analyses showed that the control variables were not directly correlated with any of the outcome variables. Thus, for reasons of parsimony and to obtain a better fitting model, I considered only the significant paths between the control variables and the outcome variables in our main models.

## Results

### Descriptive Statistics

Descriptive statistics for the measures taken on the children including the numbers of children, means, standard deviations, minimums, and maximums are shown in Table 1. The correlations between the child variables are displayed in Table 2.

**TABLE 2:** Concurrent and longitudinal correlations between child variables.

	**2.**	**3.**	**4.**	**5.**	**6.**	**7.**	**8.**	**9.**	**10.**
1. Language Background	−0.11(266)	−0.30(256)	−0.09(246)	−0.15(256)	−0.15(220)	−0.15(103)	−0.04(103)	−0.18(115)	−0.10(116)
2. HISEI		0.31 (256)	0.10 (246)	0.18 (256)	0.21 (220)	0.33 (103)	0.01 (103)	0.34 (115)	0.25 (116)
3. Language (3;6 years)		–	0.50 (245)	0.44 (255)	0.52 (214)	0.48 (101)	0.18 (101)	0.49 (113)	0.46 (114)
4. WM (3;6 years)			–	0.42 (245)	0.35 (208)	0.28 (99)	0.24 (99)	0.29 (108)	0.36 (109)
5. NON (3;6 years)				–	0.26 (213)	0.21 (100)	0.17 (100)	0.27 (112)	0.32 (113)
6. ToM (5;6 years)					–	0.28 (97)	0.03 (97)	0.35 (100)	0.24 (101)
7. MSL (9;2 years)						–	0.24 (103)	0.44 (79)	0.51 (80)
8. MK (9;2 years)							–	0.26 (79)	0.43 (80)
9. DELKO (13;7 years)								–	0.63 (115)
10. NEPS (13;7 years)									–

*N in parentheses; HISEI, Highest International Socio-Economic Index of Occupational Status in the family; WM, Working Memory; NON, Non-verbal reasoning; ToM, Theory of Mind; MSL, Mental State Language; MK, Metacognitive Knowledge; DELKO, Test for listening text comprehension; NEPS, Test for reading text comprehension.*

With regard to background characteristics it can be seen in Table 2 that whereas the family’s language background was primarily correlated with language skills (*r* = -0.30), the HISEI was also related to the other variables in our model to a moderate degree: Besides language at age 3;6 (*r* = 0.31), HISEI was related to ToM at age 5;6 (*r* = 0.21), to mental state language at age 9;2 (*r* = 0.33), and to listening comprehension (*r* = 0.34) and reading comprehension (*r* = 0.25) at age 13;7.

Foundational cognitive skills at age 3;6 were moderately related to listening and reading comprehension (*r* = 0.27–0.36), whereas descriptively the correlations with reading comprehension were slightly higher. The correlations of the foundational cognitive skills with the various facets of mental state understanding were in a small to moderate range (*r* = 0.17–0.35) and were highest for the relation between working memory and ToM.

In comparison with foundational cognitive skills, foundational language skills at age 3;6 were more strongly correlated with both listening (*r* = 0.49) and reading (*r* = 0.46) comprehension. However, language skills were also related to ToM (*r* = 0.52) and mental state language (*r* = 0.48), which were again related to listening (*r* = 0.35 and *r* = 0.44) and reading comprehension (*r* = 0.24 and *r* = 0.51). This is a first hint and prerequisite that higher-order skills in mental state understanding may mediate the relation between foundational language skills and text comprehension. However, unexpectedly, I found only a small correlation between language skills and metacognitive knowledge (*r* = 0.18), whereas metacognitive knowledge was more strongly related to reading comprehension (*r* = 0.43) than to listening comprehension (*r* = 0.26).

### Testing the DIET Model

Table 3 depicts the results for Model 1a, the complete model, which includes all direct and indirect links between the variables in the model. It shows direct relations of language skills at 3;6 to listening comprehension (β = 0.22, *p* < 0.05) but not to reading comprehension (β = 0.15, *p* = 0.19) at 13;7. Similarly, it shows direct relations of ToM at 5;6 to listening comprehension (β = 0.19, *p* < 0.05) but not to reading comprehension (β = 0.06, *p* = 0.52). By contrast, there were significant direct relations for mental state language (β = 0.31, *p* < 0.01) and metacognitive knowledge (β = 0.26, *p* < 0.01) at 9;2 with reading comprehension at 13;7, whereas mental state language was only marginally related (β = 0.18, *p* < 0.10) and metacognitive knowledge (β = 0.11, *p* = 0.25) was not related to listening comprehension at 13;7.

**TABLE 3:** Standardized regression weights between variables and model fit indices for Model 1a (general model) predicting listening comprehension and reading comprehension.

	**Listening Comprehension**				**Reading Comprehension**			
	**ToM**	**MSL**	**MK**	**LC**	**ToM**	**MSL**	**MK**	**RC**
		
	**Model 1a**				**Model 1a**			
HISEI	–	0.24**	–	0.17*	–	0.25**	–	0.05
WM	0.12^+^	0.18^+^	0.17	0.03	0.13^+^	0.21*	0.21^+^	0.04
NON	0.03	–0.02	0.05	0.02	0.03	–0.03	0.04	0.07
Language	0.45**	0.34**	0.13	0.22*	0.45**	0.33**	0.12	0.15
ToM	–	0.06	–0.06	0.19*	–	0.06	–0.06	0.06
MSL		–	–	0.18^+^		–	–	0.31**
MK		–	–	0.11		–	–	0.26**
*R*^2^	0.29	0.34	0.07	0.36	0.29	0.36	0.08	0.40

*LC, Listening Comprehension; RC, Reading Comprehension; Language, Language Skills; HISEI, Highest International Socio-Economic Index of Occupational Status in the family; WM, Working Memory; NON, Non-verbal cognitive abilities; MSL, Mental State Language; MK, Metacognitive Knowledge;^∗∗^p < 0.01, ^∗^p < 0.05, ^+^p < 0.10.*

In addition, foundational cognitive skills, i.e., working memory (β = 0.21, *p* < 0.01) and non-verbal reasoning (β = 0.09, *p* < 0.05) as well as language skills at age 3;6 (β = 0.16, *p* < 0.01) but not ToM (β = 0.00, *p* = 0.89) at 5;6 significant showed indirect relations with listening comprehension. For reading comprehension, slightly different relations were found. Whereas working memory (β = 0.12, *p* < 0.05) and language skills (β = 0.16, *p* < 0.01) at age 3;6 also showed indirect relations to reading comprehension, non-verbal reasoning (β = 0.00, *p* = 0.95) did not. Similar to the finding for listening comprehension, ToM (β = 0.00, *p* = 0.95) at age 5;6 showed no significant indirect relation to reading comprehension.

Table 4 depicts the model fits for the various models differing in the specified direct links between earlier variables and later text comprehension. The comparisons of Model 1a (complete model) with Models 1b to 1d using the BIC and AIC provided no clear results. The X ^2^-difference tests indicated that none of the restricted models differed significantly from the unrestricted complete Model 1a for reading comprehension or for listening comprehension. This means that the more restricted models should all be favored for reasons of parsimony.

**TABLE 4:** Model fit for the different models successively removing the direct links between text comprehension and ToM (Model 1b), language skills (Model 1c), and cognitive skills (Model 1d).

	**Listening Comprehension**	**Reading Comprehension**
	**Model 1a (complete)**	**Model 1a (complete)**
**MODEL FIT**		
X ^2^	X ^2^(6) = 1.56, *p* = 0.96	X ^2^(6) = 1.48, *p* = 0.96
CFI	1.00	1.00
RMSEA	0.00	0.00
AIC	6472.621	6632.638
BIC	6644.809	6804.826
	**Model 1b (ToM)**	**Model 1b (ToM)**
**MODEL FIT**		
X ^2^	X ^2^(7) = 5.27, *p* = 0.63	X ^2^(7) = 1.89, *p* = 0.97
CFI	1.00	1.00
RMSEA	0.00	0.00
AIC	6474.338	6631.044
BIC	6642.921	6650.627
	**Model 1c (Language Skills)**	**Model 1c (Language Skills)**
**MODEL FIT**		
X ^2^	X ^2^(7) = 5.09, *p* = 0.65	X ^2^(7) = 3.19, *p* = 0.87
CFI	1.00	1.00
RMSEA	0.00	0.00
AIC	6474.153	6632.343
BIC	6642.736	6800.944
	**Model 1d (Cognitive Skills)**	**Model 1d (Cognitive Skills)**
**MODEL FIT**		
X ^2^	X ^2^(8) = 1.80, *p* = 0.98	X ^2^(8) = 2.56, *p* = 0.96
CFI	1.00	1.00
RMSEA	0.00	0.00
AIC	6468.869	6629.716
BIC	6488.035	6794.729
	**Model 1e (Complete Mediation)**	**Model 1d (Complete Mediation)**
**MODEL FIT**		
X ^2^	X ^2^(10) = 15.73, *p* = 0.11	X ^2^(10) = 8.08, *p* = 0.62
CFI	1.00	1.00
RMSEA	0.00	0.00
AIC	6478.795	6631.239
BIC	6636.634	6789.078

*CFI, Comparative Fit Index; RMSEA, Root Mean Square Error of Approximation; AIC, Akaike Information Criterion; BIC, Bayesian Information Criterion.*

With regard to the complete mediation model (Model 1e) in comparison with the complete direct model (Model 1a), the X ^2^-difference test favored the less restrictive model in the case of listening comprehension [ΔX ^2^(4) = 14.17, *p* < 0.01], whereas it favored the more restrictive model for reading comprehension [ΔX ^2^(4) = 6.60, *p* = 0.16]. Also, the BIC and AIC favored the more restrictive model for reading comprehension, whereas for listening comprehension, the AIC but not the BIC favored the less restrictive model.

Altogether, it can be concluded that in the case of reading comprehension, the complete mediation model was favored, whereas in the case of listening comprehension, the direct links with foundational language and cognitive skills were also meaningful.

### Direct and Indirect Relations With Reading Comprehension via Listening Comprehension

I fit three models to the data to investigate whether listening comprehension could explain all the direct relations with reading comprehension: In Model 2a, all the direct relations of all variables with listening and reading comprehension were specified as a baseline model; in Model 2b, based on the preceding result that for reading comprehension the full mediation model (see Model 1e above) should be favored, I specified only the direct paths between reading comprehension and metacognitive knowledge as well as mental state language, whereas the direct paths from foundational cognitive as well as language skills and ToM were restricted to zero; in Model 2c (complete mediation model) all direct relations with reading comprehension were constrained to zero.

Given that non verbal reasoning did not show significant relations with text comprehension measures in the previous models (see Table 3), for reasons of parsimony, only the direct relations between non verbal reasoning, working memory, and language at age 3;6 were specified. For similar reasons (see Table 3), the direct relations of working memory with listening and reading comprehension were not specified.

**FIGURE 2:**
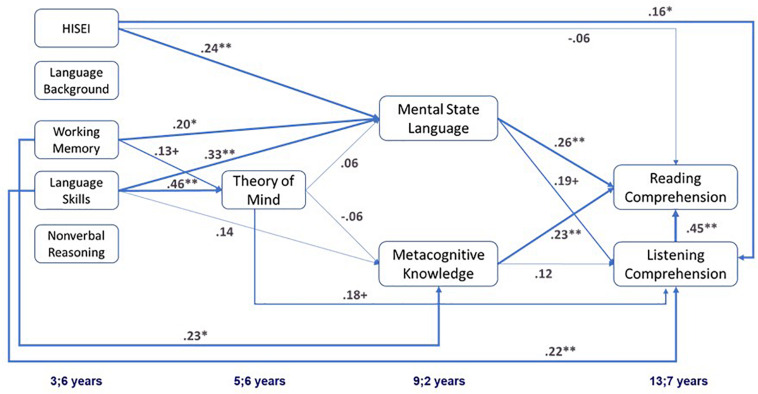
Best fitting model (Model 2b) showing the standardized equation parameters (β) for the relation between foundational cognitive and language skills, facets mental state understanding and, listening as well as reading comprehension. **p < 0.01, *p < 0.05, ^+^*p* < 0.10.

Table 5 shows the standardized beta weights for all three specified models. All model fit indicators agreed that the fit of Model 2b was superior to the fit of the less restrictive Model 2a [ΔX ^2^(3) = 1.06, *p* = 0.78] as well as the fit of the more restrictive Model 2c [ΔX ^2^(2) = 22.02, *p* < 0.01]. Thus, Model 2b was chosen as the final model (see Figure 2).

**TABLE 5:** Standardized regression weights between variables and model fit indices for Model 2a to Model 2c for predicting reading comprehension (RC).

	**Model 2a**					**Model 2b**					**Model 2c**				
	**ToM**	**MSL**	**MK**	**LC**	**RC**	**ToM**	**MSL**	**MK**	**LC**	**RC**	**ToM**	**MSL**	**MK**	**LC**	**RC**
HISEI	–	0.24**	–	0.16*	–0.02	–	0.24**	–	0.16*	–	–	0.24**	–	−0.16*	–
WM	0.13^+^	0.20*	0.23*	–	–	0.13^+^	0.20*	0.23*	–	–	0.13^+^	0.17^+^	0.19^+^	–	–
Language	0.46**	0.32**	0.13	0.23*	0.09	0.46**	0.33**	0.14	0.22*	–	0.46**	0.33**	0.14	0.23*	–
ToM		0.06	–0.06	0.18^+^	–0.02		0.06	–0.06	0.18^+^	–		0.06	–0.06	0.18^+^	–
MSL				0.19^+^	0.23**				0.19^+^	0.26**				0.19^+^	–
MK				0.12	0.22**				0.12	0.23**				0.11	–
LC					0.44**					0.45**					0.63**
X ^2^	X ^2^(14) = 3.57, *p* = 0.99					X ^2^ (17) = 4.63, p = 0.99					X ^2^(19) = 26.65, *p* = 0.11				
CFI	1.00					1.00					0.97				
RMSEA	0.00					0.00					0.04				
AIC	7145.012					7140.072					7158.09				
BIC	7327.961					7312.260					7323.11				
*R*^2^	0.29**	0.36**	0.09	0.35**	0.52**	0.29**	0.36**	0.09	0.35**	0.51**	0.29**	0.34**	0.07	0.35**	0.39**

*LC, Listening Comprehension; RC, Reading Comprehension; Language, Language Skills; WM, Working Memory; MSL, Mental State Language; MK, Metacognitive Knowledge; HISEI, Highest International Socio-Economic Index of Occupational Status in the family; CFI, Comparative Fit Index; RMSEA, Root Mean Square Error of Approximation; AIC, Akaike Information Criterion; BIC, Bayesian Information Criterion. ^∗∗^p < 0.01, ^∗^p < 0.05, ^+^p < 0.1.*

Model 2b shows that mental state language and metacognitive knowledge at age 9;2 were both significantly related to reading comprehension at age 13;7 beyond listening comprehension assessed at the same time point. With regard to indirect relations, working memory as a foundational cognitive skill had only a marginally significant indirect relation with later reading comprehension via mental state language (β = 0.05, *p* < 0.10) and metacognitive knowledge (β = 0.05, *p* < 0.10). By contrast, early language skills showed various significant indirect relations with reading comprehension: (a) via mental state language (β = 0.08, *p* < 0.05), (b) via listening comprehension (β = 0.10, *p* < 0.05), and (c) via ToM and listening comprehension (β = 0.04, *p* < 0.10). However, this last one was only a marginally significant indirect relation. ToM also showed a marginally significant indirect relation with reading comprehension via listening comprehension (β = 0.08, *p* < 0.10). In addition, there was a marginally significant indirect relation between mental state language and reading comprehension via listening comprehension (β = 0.09, *p* < 0.10).

## Discussion

The main aim of this study was to test a part of the DIET model by focusing on the roles of different facets of mental state knowledge and understanding and their relations to language and text comprehension. I extended the DIET model by adding metacognitive knowledge and mental state language as additional higher-order skills to the model and fit it to longitudinal data from ages 3;6 to 13;7. Our results supported the main ideas of the DIET model (Kim, 2017) by showing that early foundational language skills (as well as working memory) were indirectly related to text comprehension via higher-order skills such as ToM, mental state language, and listening comprehension. However, our study added new and partly unexpected findings.

First, I found different relational patterns between early foundational language skills, facets of mental state knowledge and understanding, and text comprehension in early adolescence for listening comprehension in contrast to reading comprehension. Relatedly, listening comprehension did not mediate the relations of higher-order skills, namely, mental state language and metacognitive knowledge, with reading comprehension.

Second, our study revealed that ToM was only weakly associated with advanced text comprehension, especially with reading comprehension, and had no indirect relations to text comprehension via advanced measures of mental state understanding, namely, metacognitive knowledge and mental state language.

I discuss these findings in more detail below.

### Different Relational Patterns for Listening and Reading Comprehension

Although listening and reading comprehension in early adolescence should be strongly related and influenced by similar predictors because reading comprehension at this age is less constrained by decoding processes (Foorman et al., 2015), I found different relational patterns with regard to early foundational cognitive and language variables as well as higher-order skills that are related to mental state knowledge and understanding. First, whereas language skills and ToM in preschool showed direct relations with listening comprehension in early adolescence, there were no direct relations between early foundational skills or ToM with reading comprehension in early adolescence. Accordingly, for reading comprehension but not for listening comprehension, a complete mediation model without any direct relations of foundational skills fit best. Second, metacognitive knowledge and mental state language were more strongly related to reading comprehension than to listening comprehension and showed direct relations with reading comprehension even after listening comprehension was controlled for.

Why do language skills and ToM have direct relations with listening comprehension but not with reading comprehension? And why are metacognitive knowledge and mental state language more strongly related to reading comprehension than listening comprehension? To answer these questions, it might be helpful to take a look at the differences between reading and listening comprehension: Whereas listening comprehension refers to the comprehension of orally presented text, reading comprehension refers to the comprehension of written text. Even if decoding processes play only a minor role in reading comprehension in advanced reading, different information processing is probably at work. For example, given that a person can reread a written text but not an orally presented text, working memory might be more important for listening comprehension than reading comprehension. In this vein, Roch et al. (2012) demonstrated that people with Down syndrome show better reading comprehension than listening comprehension and that verbal memory contributes to explaining this advantage of reading over listening comprehension. Though, our study showed that early working memory in preschool does not have direct relations on either listening or reading comprehension and might play a similar role in the two. However, to confirm that different information processes are at work in reading and listening comprehension in early adolescence, it would be necessary to control for concurrent information processing skills.

The idea that listening and reading comprehension may require different processes even in early adolescence was supported by the result that metacognitive knowledge and mental state language showed direct relations with reading comprehension over and above listening comprehension and that metacognitive knowledge and mental state language are not or are only slightly related to listening comprehension. Therewith, our results suggest that listening comprehension is not the only variable that explains variance in advanced reading comprehension when the constraints of decoding processes are small: Higher-order processes such as metacognitive knowledge and mental state language additionally contribute to reading success in the later school years. This finding leads to the suggestion that children might profit from written text comprehension beyond their (oral) language comprehension skills when they possess metacognitive knowledge and when they are advanced in understanding mental state language. This implication also reflects the common knowledge that some people are better at comprehending spoken language, whereas others are better with written language.

Metacognitive knowledge and mental state language are more strongly related to reading comprehension than they are to listening comprehension. This suggests that these skills are especially helpful for comprehending written but not orally presented texts. Thus, our results suggest that metacognitive knowledge and mental state language might provide a means for facilitating written text comprehension, especially when oral text comprehension is low. Those children who comprehend written texts well may possess and use knowledge about learning and reading, i.e., metacognitive knowledge or knowledge about mental state words, to do well in reading comprehension tasks, even when their oral listening comprehension is low (see also Roch et al., 2012). Thus, it seems likely that especially poor language comprehenders may profit from metacognitive training programs (for a similar argument see Kendeou et al., 2007; Oakhill et al., 2019).

Different from the present study’s results, Kim (2017) showed that listening comprehension completely mediated the relation between higher-order skills and reading comprehension. However, in contrast to Kim (2017), I assessed different higher-order skills, namely, those that are specifically related to mental state understanding, and I assessed foundational skills, higher-order skills, and text comprehension skills at different points in development.

Although the present study supports the conclusion that the development of reading and listening comprehension is influenced by different developmental variables I could not prove causality with this study design. To do so, at least repeated measures of reading and listening comprehension during development are necessary. Only with such measures would it be possible to tell whether earlier variables have a different impact on the change in reading and listening comprehension.

In addition, I did not control for decoding processes in my study. Thus, differences in decoding processes could be responsible for the direct relations of metacognitive knowledge and mental state language with reading comprehension. In contrast to our findings, Lervåg et al. (2018) found that listening comprehension and word reading at 7.5 years of age explained almost all of the variability in reading comprehension up to the age of 12.5 years. A possible reason for why Lervåg et al. (2018) explained such a large amount of variance in reading comprehension through listening comprehension may reflect the fact that they were able to use a latent variable account and could eliminate problems due to measurement errors. Lervåg et al. (2018) suggested that measurement errors are a reason for the discrepancy between studies that found differentiated effects of early language and cognitive skills beyond listening comprehension. However, Lervåg et al. (2018) assessed listening comprehension and language measures at the age of 7.5 years. By contrast, we assessed listening comprehension along with reading comprehension in adolescence and basic language skills and working memory at earlier points in time. As in Lervag et al.’s study, early language and cognitive skills did not impact reading development beyond listening comprehension in the present study. Thus, it is possible that early listening comprehension is a better predictor of advanced reading comprehension than listening comprehension assessed at the same time. It might be that when the contents of the listening comprehension tasks become more complicated, listening comprehension is less relevant for reading comprehension, and other factors such as metacognitive processes or specific language skills become more critical.

Another explanation for the different relational patterns between language, ToM, and listening comprehension in contrast to those between language, ToM, and reading comprehension might lie in the similarities between the first set of measures: ToM tasks as well as foundational language tasks are both listening comprehension tasks. Thus, the direct relations between language and listening comprehension as well as between ToM and listening comprehension might be a simple method effect. However, this still does not explain why I also found relations for advanced measures of mental knowledge and understanding with reading comprehension that could not be explained by listening comprehension. Thus, it is very likely that listening comprehension and reading comprehension are indeed differently predicted by earlier skills and require different informational processes.

### The Relation Between Early ToM and Text Comprehension in Early Adolescence

The present study also adds to our knowledge about the relation between ToM and reading comprehension. In contrast to most of the earlier studies that investigated the relation between ToM and reading comprehension, we measured ToM in preschool and reading comprehension in early adolescence and thus investigated a much longer period of time. I proposed that the non-significant relations between ToM and reading comprehension found in previous studies (Guajardo and Cartwright, 2016; Lockl et al., 2017) could be explained by the fact that the children were in the early stages of learning to read. Although these studies were longitudinal, none of them followed children over an extended period such as until early adolescence when children’s reading skills become more advanced. I expected that ToM would be more relevant for advanced reading comprehension than for reading comprehension in the early stages when reading comprehension is constrained by decoding processes and the texts that have to be comprehended are easy and do not require much reasoning about mental states. However, the relation I found between ToM at age 5 and reading comprehension 8 years later was small, and after considering other relevant variables in our model, there were no direct relations between ToM and reading comprehension.

I suggest different explanations for this result. First, because of the close relation between language and ToM (Astington and Baird, 2005a), it might not be possible to separate the effects of early language skills and ToM. In another study with a different focus but almost the same data set, I controlled for language skills assessed at the same measurement point as ToM and showed that the relation between ToM and later reading comprehension as well as listening comprehension decreased even more (Ebert, 2020). Thus, the effects of ToM may primarily be driven by the variance that it shares with language competencies, and as soon as language skills are considered, ToM might not be uniquely related. However, I found a unique relation of early ToM and language skills with later listening comprehension. This finding is in accordance with other studies (Kim, 2015; Guajardo and Cartwright, 2016) that also reported indirect relations with reading comprehension via listening comprehension. This suggests that at least for advanced listening comprehension, ToM might be uniquely related beyond early language skills.

Another explanation for why I did not find a strong relation between ToM and later text comprehension measures, especially reading comprehension, may lie in the ToM measure that was used in the study. False belief understanding was assessed at a relatively late time point in development when most children should already understand first-order false belief tasks (Wellman et al., 2001). Thus, differences in ToM in our preschool measure may reflect only whether children can master second-order false belief tasks, which may not be as relevant for text comprehension as a metarepresentational ToM understanding. However, the children are not at the ceiling and show quite a variability in their ToM understanding. Moreover, Kim (2017) also reports a correlation between reading comprehension and second-order false belief understanding. Thus, it is not to be expected that only first-order false belief understanding would be related to reading comprehension. Moreover, other studies suggest that in particular, more advanced measures of ToM are stronger related to text comprehension than first-order false belief understanding (e.g., Boerma et al., 2017; Ebert, 2020; Florit et al., 2020).

A third explanation for why I did not find a relation between ToM and reading comprehension after controlling for foundational cognitive and language skills may lie in the fact that in contrast to previous studies, reading comprehension was assessed at a much later time in development than the other studies did. It is possible that at a certain point in advanced reading comprehension, ToM may be less strongly related to reading comprehension because other variables facilitate reading comprehension more than listening comprehension. Different from what the simple view of reading suggests, listening comprehension might not be the only factor that contributes to more advanced reading comprehension (see also Kirby and Savage, 2008). Indeed, when only a direct relation of listening comprehension with reading comprehension is allowed, and all other direct paths are constrained to zero, listening comprehension explains 40% of the variance in reading comprehension. Thus, there is still much variance to be explained in advanced reading comprehension. Moreover, our study revealed an additional effect of metacognitive knowledge and mental state language on reading comprehension, which was not explained by listening comprehension, earlier foundational cognitive and language skills, or ToM. Thus, in advanced reading comprehension, or at least in our measure of reading comprehension, as already discussed above, additional skills may be helpful for reading comprehension.

### Advanced Measures of Mental State Understanding as Mediating Factors

Whereas mental state language mediated the relations between early language skills and later text comprehension, metacognitive knowledge did not. This was mainly explained by the fact that, unexpectedly, neither early language skills nor ToM were related to metacognitive knowledge during the school years. This finding also suggests that early language and ToM may only be relevant for the early steps in developing an understanding of the mental world and building one’s initial knowledge about mental states and processes (Ebert, 2011, 2015). However, after developing a basic mental understanding or a representational understanding of the mind, amassing factual knowledge about the mind may require different sources. For example, instructional processes and learning experiences may become more critical at this point. This assumption might also explain why early ToM was also not related to later mental state language, although a conceptual overlap between specific language knowledge about mental states and knowledge about mental states was assumed (e.g., Astington and Pelletier, 2005; Bretherton and Beeghly, 1982). Thus, our results show that general language skills may be more important for how well children understand the specific meaning of mental verbs a few years later than whether they have developed a representational understanding of the mind early.

### Early Language and Text Comprehension in Early Adolescence

Our study also demonstrates that early language skills are related to later reading comprehension in many ways, although there are no direct relations beyond listening comprehension relations on reading comprehension (for similar results, see Kim, 2015; Lervåg et al., 2018). However, early language skills showed a small indirect relation via ToM, which was again related to listening comprehension and this again was strongly related to reading comprehension. Moreover, early language skills were also related to listening comprehension directly beyond the relations with working memory, ToM, and mental state language. In addition, early language skills also had indirect relations with reading comprehension via mental state language. Mental state language can again also be interpreted as a listening comprehension task with a specific mental content. Against this background, our findings suggest that the skills that are necessary for comprehending oral texts, no matter whether they are about specific mental terms or contain more general content, explain the relation between early language skills and advanced reading comprehension. Also in support of this idea, in a study using almost the same data set, I found that the Strange Stories (White et al., 2009) – which are another advanced measure of ToM and can also be interpreted as a listening comprehension task with a specific focus on mental states – were also related to reading comprehension (Ebert, 2020). A similar result was found in a recent study by Florit et al. (2020), which showed that children’s advanced ToM is a unique predictor of multiple-text comprehension.

Thus, an important conclusion of our study is that early language skills have a long-lasting impact on further development and that they impact later text comprehension and particularly reading comprehension in many ways.

To sum up, our study demonstrated the importance of early language, ToM, mental state language, and metacognitive knowledge for children’s later reading comprehension. However, many open questions about how understanding of the mental world (ToM), factual knowledge about strategies, memory or learning processes (metacognitive knowledge), and the language related to mental state understanding are related over time and how they contribute to children’s reading development and general educational development along with general language competencies remain (see also Hughes and Devine, 2015; Lockl et al., 2017). In addition, early language skills may also support attentional processes and higher-order skills such as inference making skills or comprehension monitoring that are necessary for oral comprehension, no matter whether it is a specific mental language comprehension task or a more general one. In the present study, I focused on only higher-order skills that are related to mental state understanding. However, previous studies found that attentional processes and other higher-order skills could also explain variance in reading comprehension (e.g., Silva and Cain, 2015; Kim, 2016). Thus, it is up to future studies to include all the facets of higher-order skills and further increase the knowledge about the development of reading comprehension. In particular, more research including repeated measures of listening and reading comprehension are desirable for gathering knowledge about the impact of the various components on developmental trajectories.

## Data Availability Statement

The datasets generated for this study are available on request to the corresponding author.

## Ethics Statement

Ethical review and approval was not required for the study on human participants in accordance with the local legislation and institutional requirements. Written informed consent to participate in this study was provided by the participants’ legal guardian/next of kin.

## Author Contributions

The author confirms being the sole contributor of this work and has approved it for publication.

## Conflict of Interest

The authors declare that the research was conducted in the absence of any commercial or financial relationships that could be construed as a potential conflict of interest.

## Appendix

### Example Story – Comprehension of Mental Verbs

Petra is watching a movie on TV. The movie shows a bank robbery. Suddenly there is thunderstorm and the movie is interrupted. Petra thinks that the gardener robbed the bank. At the next day Petra meets Tim. Tim has watched the whole movie. He says to her that the gardener robbed the bank. Does Tim concede, confirm, or consider that the gardener has robbed the bank?

Target mental verbs of all stories: German equivalent for conclude (daraus schließen), confirm (bestätigen), assume (davon ausgehen), predict (vorhersagen), expect (erwarten), concede (einräumen), infer (daraus folgern), deliberate (abwägen), assert (behaupten), presume (annehmen), insure (versichern), imply (voraussetzen), consider (überlegen), deny (bestreiten).
